# Robotic *Versus* Laparoscopic Distal Pancreatectomy for Pancreatic Ductal Adenocarcinoma: A Systematic Review and Meta-Analysis

**DOI:** 10.3389/fonc.2021.752236

**Published:** 2021-09-20

**Authors:** Qingbo Feng, Chuang Jiang, Xuping Feng, Yan Du, Wenwei Liao, Hongyu Jin, Mingheng Liao, Yong Zeng, Jiwei Huang

**Affiliations:** ^1^Department of Liver Surgery and Liver Transplantation Centre, West China Hospital, Sichuan University, Chengdu, China; ^2^Department of Liver Surgery, The First Clinical Medical College of Lanzhou University, Lanzhou, China

**Keywords:** distal pancreatectomy, pancreatic ductal adenocarcinoma, robotic, laparoscopic, meta-analysis

## Abstract

**Background:**

Robotic distal pancreatectomy (RDP) and laparoscopic distal pancreatectomy (LDP) are the two principal minimally invasive surgical approaches for patients with pancreatic body and tail adenocarcinoma. The use of RDP and LDP for pancreatic ductal adenocarcinoma (PDAC) remains controversial, and which one can provide a better R0 rate is not clear.

**Methods:**

A comprehensive search for studies that compared robotic *versus* laparoscopic distal pancreatectomy for PDAC published until July 31, 2021, was conducted. Data on perioperative outcomes and oncologic outcomes (R0-resection and lymph node dissection) were subjected to meta-analysis. PubMed, Cochrane Central Register, Web of Science, and EMBASE were searched based on a defined search strategy to identify eligible studies before July 2021.

**Results:**

Six retrospective studies comprising 572 patients (152 and 420 patients underwent RDP and LDP) were included. The present meta-analysis showed that there were no significant differences in operative time, tumor size, and lymph node dissection between RDP and LDP group. Nevertheless, compared with the LDP group, RDP results seem to demonstrate a possibility in higher R0 resection rate (p<0.0001).

**Conclusions:**

This systematic review and meta-analysis suggest that RDP is a technically and oncologically safe and feasible approach for selected PDAC patients. Large randomized and controlled prospective studies are needed to confirm this data.

**Systematic Review Registration:**

https://www.crd.york.ac.uk/PROSPERO/#recordDetails, identifier [CRD42021269353].

## Introduction

The incidence of pancreatic cancer has risen and is likely to become the second most frequent cause of cancer-related death by 2030 (1). Pancreatic duct adenocarcinoma (PDAC) is the most common type of pancreatic cancer and is usually located in the head of the pancreas (2). Distal pancreatectomy is the fundamental surgery for the treatment of body-tail tumors of the pancreas. Since Cuscheri et al. reported the first laparoscopic distal pancreatectomy (LDP) in 1996 (3), and Melvin et al. performed the first robotic distal pancreatectomy (RDP) in 2003 (4). RDP and LDP applied to the surgical treatment of pancreatic tumors have increased over the last decade. Thanks to 3D high-definition visualization, tremor filtration, and instrument dexterity of robotic surgery systems, robotic surgery has emerged as a viable alternative approach to conventional laparoscopic surgery (5). Some literatures have confirmed the safety and feasibility of RDP and emphasized its advantages in less bleeding, lower conversion rate, shorter hospital stay, and higher spleen preservation rate (6–9). These studies involved patients with pancreatic ductal adenocarcinoma, pancreatic neuroendocrine tumor, intraductal papillary mucinous neoplasm, solid pseudopapillary tumor, or some benign tumors; therefore, RDP and LDP, which one is the better approach for PDAC, is unclear. To the best of our knowledge, no system review and meta-analysis has been performed to analyze the perioperative short-term oncological outcomes of minimally invasive distal pancreatectomy (RDP and LDP) for PDAC. This systematic review and meta-analysis aimed to compare the perioperative and oncologic outcomes of RDP *versus* LDP for PDAC.

## Methods

### Data Sources and Search Strategy

This study has registered at PROSPERO, and registration number is CRD42021269353 and reported on the basis of the PRISMA guidelines (10). Studies that investigated RDP *versus* LDP for PDCA were systematically searched in PubMed, Web of Science, EMBASE, Cochrane Central Register, and ClinicalTrials.gov databases before July 31, 2021, by two independent investigators (QF, CJ). The search terms used were “robotic surgery,” “laparoscopic surgery,” “distal pancreatectomy,” “left pancreatectomy,” “pancreatic cancer,” “pancreatic ductal adenocarcinoma,” and “adenocarcinoma,” either individually or in combination. The “related articles” function was used to broaden the search, and all citations were considered for relevance. Manual search of the references of publication was adopted to prevent missing relevant researches.

### Inclusion and Exclusion Criteria

Two investigators (QF, CJ) reviewed currently available literature and screened all titles and abstracts independently and identified eligible studies according to the following criteria.

Inclusion criteria were as follows: (1) Participants: patients with pancreatic body and tail adenocarcinoma, and PDAC was defined by histologically; (2) Types of interventions: RDP and LDP; (3) Study type: randomized controlled trials (RCTs), propensity score matching studies, retrospective studies, cohort studies, and case-control studies comparing RDP to LDP with PDAC patients; (4) At least one outcome was reported in the literature, including operation time, intraoperative bleeding, tumor size, R0 rate, conversion rate, lymph node harvested, and spleen preservation rate; (5) Language restrictions: English.

Exclusion criteria were the following: (1) Conference abstracts, editorials, letters, case reports; (2) No comparative analysis between RDP and LDP.

### Data Extraction and Quality Assessment

The original data from all candidate articles were independently assessed and extracted by two reviewers (QF, CJ) by using a unified datasheet, and any ambiguity was resolved by a third researcher (XF). The major data extraction includes the following: name of first author, publication year, study design, country, number of patients, mean age, gender, operative times, tumor size, bleeding, hospitalization, overall complication, overall complications, mortality, blood transfusion, R0 rate. The quality of the eligible studies was assessed by Newcastle-Ottawa Scale (NOS) by two different assessors (11). Every included study was independently evaluated by two authors (QF, XF), and NOS score ≥6 is considered as being of high quality.

### Statistical Analysis

The Review Manager 5.3 software was used for statistical analyses. The 95% confidence interval (CI) and mean difference (MD) were used for continuous data, while categorical variables using odds ratio (OR). The method originally described by Hozo et al. to convert medians with ranges into means with standard deviations was used (12). Potential publication bias was visually assessed by Begg’s funnel plot and Egger’s test. Statistical heterogeneity was quantified using I^2^ value. A fixed-effects model (FEM) was adopted when heterogeneity was low or moderate (I^2^ <50%), and when heterogeneity was high (I^2^ ≥50%), a random-effects model (REM) was used.

## Results

### Characteristics of the Included Studies

**Figure 1:**
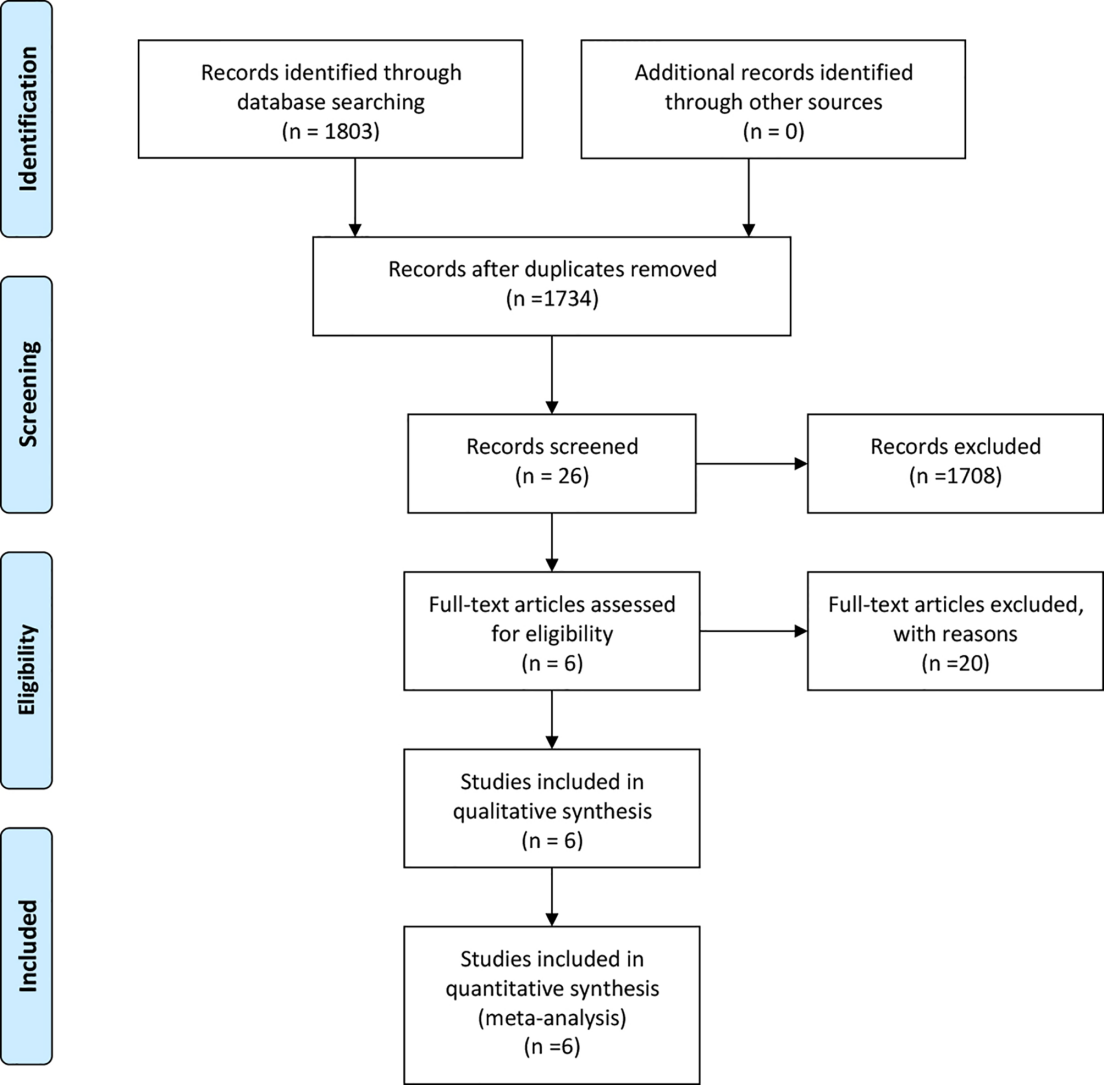
Flow chart of study identification and selection.

Finally, a total of 1,734 relevant English publications from the various electronic databases were yielded. According to the inclusion criteria, six retrospective studies (13–18) comparing RDP and LDP in a total of 572 patients (152 and 420 underwent RDP and LDP, respectively) were included for further analysis. A flow diagram of our analysis protocol is shown in **Figure 1**. The general information and summary of NOS scores of all the included studies are given in **Table 1**.

**Table 1:** The main characteristics and NOS of the included studies.

Author-year	Country	Study type	Period	RPD(n)	LPD(n)	RPD-age	LPD-age	RPD M/F	LPD M/F	NOS
Daouadi-2013 (13)	USA	Retrospective	2004–2011	13	14	NA	NA	NA	NA	7
Lee-2014 (14)	USA	Retrospective	2000–2013	4	19	NA	NA	NA	NA	8
Qu-2018 (15)	China	PSM	2011–2015	35	35	58.1 ± 11.1	57.8 ± 11.4	22/13	22/13	9
Lyman-2018 (16)	USA	Retrospective	2008–2017	23	35	63.9 ± 12.7	66.7 ± 9.1	12/11	23/12	8
Hong-2019 (17)	South Korea	Retrospective	2015–2017	12	76	57.3 ± 11.9	64.7 ± 9.5	8/4	33/43	7
Lof-2021 (18)	Italy	Retrospective	2011–2019	65	241	NA	NA	NA	NA	7

RDP, robotic distal pancreatectomy; LDP, laparoscopic distal pancreatectomy; M/F, male/female; SD, standard deviation; NOS, Newcastle-Ottawa Scale; NA, not applicable.

### Perioperative Outcomes

#### Operative Time

**Figure 2:**
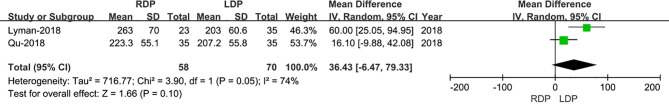
Forest plot of comparison of RDP *versus* LDP for operative time.

Only two studies (15, 16) that encompassed 128 patients (58 and 70 underwent RDP and LDP, respectively) reported operative times. The meta-analysis showed no difference in operative time in the two groups (WMD: 36.43 min; 95% CI −6.47 to 79.33; p=0.10). Heterogeneity was high (I^2 =^ 74%) and analyzed in REM (**Figure 2**).

### Postoperative Outcomes

#### Tumor Size

**Figure 3:**
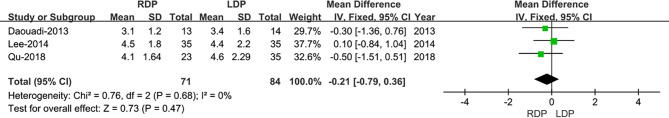
Forest plot of comparison of RDP *versus* LDP for tumor size.

Three studies (13, 15, 16) that encompassed 155 patients (71 and 84 underwent RDP and LDP, respectively) recorded the tumor size, and pooled data didn’t show any differences in tumor size in the two approaches (WMD: −0.21; 95% CI −0.79 to 0.36; I^2 =^ 0%, p =0.47) (**Figure 3**).

### Short−Term Oncological Outcomes

#### R0 Resection Rate

**Figure 4:**
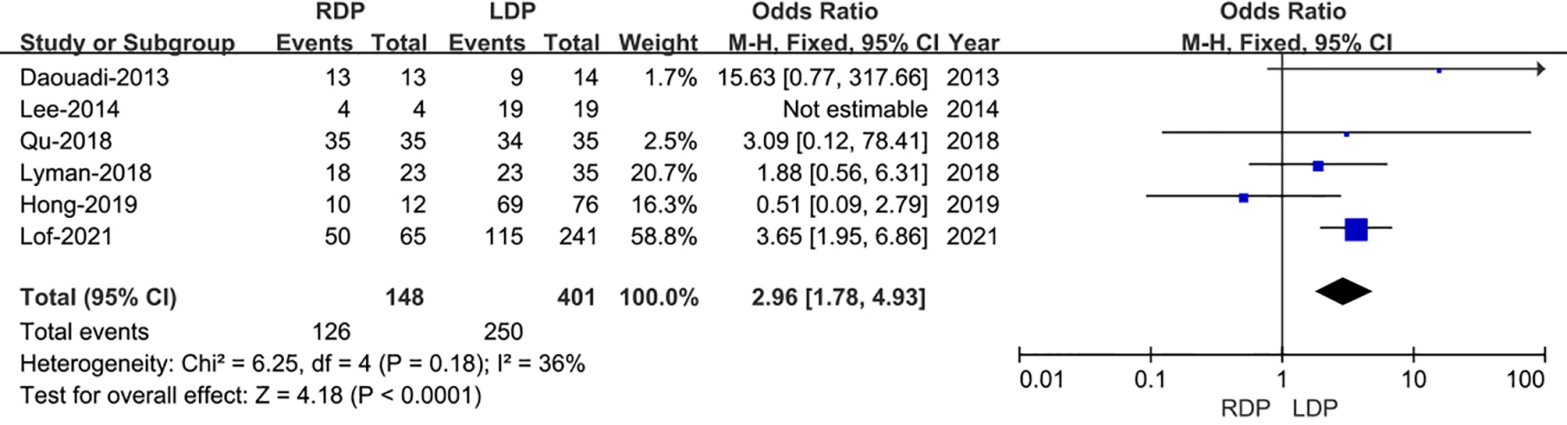
Forest plot of comparison of RDP *versus* LDP for R0 rate.

Regarding R0 resection rate, data were provided in all the six studies including 572 patients (13–18). And a meta-analysis of these data suggested that RDP was associated with a higher R0 resection rate (OR: 2.96; 95% CI 1.78–4.93; I^2 =^ 36%, p<0.0001) as shown in the FEM (**Figure 4**).

#### Lymph Node Dissection

**Figure 5:**
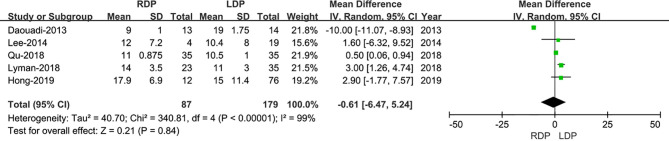
Forest plot of comparison of RDP *versus* LDP for lymph node dissection.

Five studies (13–17) assessed the number of lymph node dissection. These eight studies had great heterogeneity (I^2^ = 99%), and therefore, the REM was used. The results revealed no difference in lymph node dissection (WMD: −0.61; 95% CI −6.47 to 5.24; p= 0.84) (**Figure 5**).

### Long−Term Oncological Outcomes

#### Long-Term Survival

Only two studies (15, 17) reported OS and DFS of RDP and LDP in the treatment of PDAC. Qu et al. (15) compared the survival data after PSM of 70 patients with PDAC (35 underwent RDP and 35 underwent LDP) from China and suggested that RDP and LDP can achieve a median overall survival of 27 and 25 months, respectively (p = 0.15), and the median disease-free survival (11 months *vs.* 11 months, respectively, p = 0.25). The largest overall survival outcomes data of RDP and LDP in the treatment of PDAC comes from Korea. Hong et al. reported 88 patients with PDAC underwent RDP or LDP (12 underwent RDP and 76 underwent LDP) and revealed a non-significant difference in median OS (not reached *vs.* 32.1 months, p = 0.359) and disease-free survival (11.9 *vs.* 14.6 months, p = 0.381) (17).

### Publication Bias

**Figure 6:**
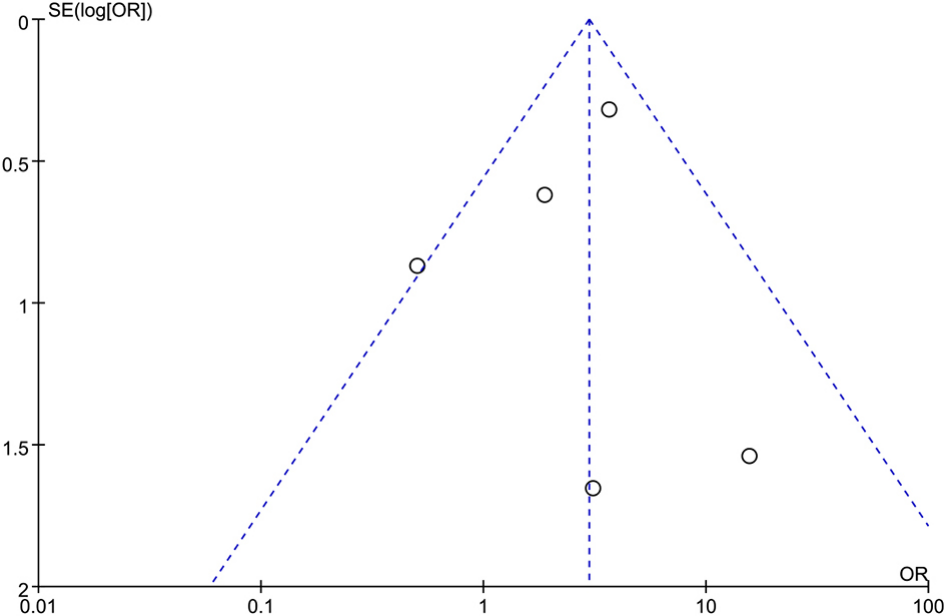
Funnel plots for R0 rate.

Funnel plot of R0 resection rate was drawn to investigate the potential publication bias. All of the studies lie inside the 95% Cis, and the funnel plot of R0 rate indicated no obvious publication bias (**Figure 6**).

## Discussion

As a standard surgical method for the treatment of benign and malignant diseases of the body and tail of the pancreas, the development of distal pancreatectomy has experienced laparotomy, laparoscopy, and robot surgery. Previous investigations indicate that LDP has a shorter length of stay, less blood loss, less pain, earlier oral intake, and faster recovery in comparison with open distal pancreatectomy (19–21). As a result of the robotic system providing better visualization and reducing natural tremors, many researchers believe that robotic surgery system can conquer the technical limitations of LDP and thus potentially provide better oncological outcomes. Despite that there have been published several other meta-analyses assessing surgical and oncological outcomes between two minimally invasive techniques of distal pancreatectomy, there are no studies focusing on PDAC (22–25). To compare the real difference between RDP and LDP in the treatment of PDAC, we analyzed the data from the literature that cases were pathologically diagnosed as PDAC. To the best of our knowledge, this is the first meta-analysis that compares RDP and LDP for patients with PDAC. The present study included 572 PDAC patients (152 and 420 patients underwent RDP and LDP, respectively). In short, this meta-analysis did not detect any statistically significant differences in operative time, tumor size, and lymph node dissection. However, the R0 resection rate was significantly higher in the RDP group than in the LDP group (85.13 *vs.* 62.34%; p<0.0001).

Surgical margins and lymph node dissections are two important malignancy prognosis factors in distal pancreatectomy. In terms of oncologic outcome, pooled data of this meta-analysis revealed that RDP has a higher rate of R0 rection than LDP. We think that this may be explained by patients with PDAC in early stage who were selected to perform RDP. From the perspective of tumor radical effect, the results of this study show that the two surgical methods have the same effect in the number of lymph node dissections, suggesting that RDP and LDP have the same tumor radical effect, which is consistent with the results of most existing clinical studies.

When it comes to long-term survival, according to our search, there are still no RCTs comparing the long-term survival between RDP to LDP in patients with PDAC. The largest overall survival outcome data of RDP and LDP in the treatment of PDAC comes from Korea. Hong et al. reported 88 patients with PDAC underwent RDP or LDP (12 underwent RDP and 76 underwent LDP) and revealed a non-significant difference in median OS (not reached *vs.* 32.1 months, p = 0.359) and disease-free survival (11.9 *vs.* 14.6 months, p = 0.381) (17). But Qu et al. (15) compared the survival data after PSM of 70 patients with PDAC (35 underwent RDP and 35 underwent LDP) from China and suggested that RDP and LDP can achieve a median overall survival of 27 and 25 months, respectively (p = 0.15), and the median disease-free survival (11 months *vs.* 11 months respectively, p = 0.25). In some ways, the pooled data demonstrated that RDP is not ontologically inferior to LDP and can even achieve superior oncologic outcomes compared to LDP.

The conversion rate, overall and major complications rate, pancreatic fistula, spleen preservation rate, and costs during RDP and LDP for PDAC were not analyzed due to data being unavailable in these studies. But Kamarajah’s study that included 3,112 patients only focuses on safety of RDP and shows no significant differences in overall and major complications, overall and high-grade pancreatic fistula, and compared to LDP, RDP was associated with lower conversion rate (23). And Guerrini’s meta-analysis shows RDP was associated with higher spleen preservation rate than LDP (24).

To evaluate the safety and efficiency of RDP for PDAC, this meta-analysis included six studies and showed that RDP was comparable to LDP. However, this review has some limitations that should be considered. First, most of the included studies were retrospective research, and there were no RCTs, which may have contributed to selection bias. Furthermore, of the six included studies, the TNM stage, tumor size, and differentiation degree of patients with PDAC have not been reported in some studies. What’s more, few studies reported long-term survival outcomes such as overall survival and 3-year survival time of RDP. Therefore, further studies, in particular large-scale prospective studies and RCTs, are expected to assess the effectiveness and safety of RDP for patients with PDAC.

In conclusion, this system review and meta-analysis suggests that RDP is a technically and oncologically safe and feasible approach for PDAC patients and seems to provide a better R0 rate. Large randomized and controlled prospective studies are needed to confirm the superiority of RDP.

## Data Availability Statement

The raw data supporting the conclusions of this article will be made available by the authors, without undue reservation.

## Author Contributions

QF, CJ, and WL: Study concept and design. QF and CJ: Literature review. QF, XF, and HJ: Statistical analysis. QF, ML, and YD: Draft of the manuscript and preliminary revision. YZ and JH: Study supervision and final approval. All authors contributed to the article and approved the submitted version.

## Funding

This work was supported by grants from the National Key Technologies R&D Program (2018YFC1106800), the Natural Science Foundation of China (82070644, 82002572, 82002967, 81972747, 81872004, 81800564, 81770615, 81700555, and 81672882).

## Conflict of Interest

The authors declare that the research was conducted in the absence of any commercial or financial relationships that could be construed as a potential conflict of interest.

## Publisher’s Note

All claims expressed in this article are solely those of the authors and do not necessarily represent those of their affiliated organizations, or those of the publisher, the editors and the reviewers. Any product that may be evaluated in this article, or claim that may be made by its manufacturer, is not guaranteed or endorsed by the publisher.
